# Supplementary material for: Changes in individuals’ glaucoma progression velocity after IOP-lowering therapy: A systematic review
Source: PLoS One. 2025 May 29;20(5):e0324806. doi: 10.1371/journal.pone.0324806 (PMC12121737; doi:10.1371/journal.pone.0324806)
Supplement: S1 File — (PDF) [file pone.0324806.s001.pdf]

Master List of Studies

| | Title | Authors | Inclusion Status | Comments |
|---|---|---|---|---|
| | Pressure or progression? | Burnstein, Y; Robin, AL | Excluded | Insufficient progression velocity data |
| | Factors for progression and glaucoma treatment: the Early Manifest Glaucoma Trial | Leske, MC; Heijl, A; Hyman, L; Bengtsson, B; Komaroff, E | Excluded | Insufficient progression velocity data |
| | Risk factors for visual field progression in the low-pressure glaucoma treatment study | De Moraes, CG; Liebmann, JM; Greenfield, DS; Gardiner, SK; Ritch, R; Krupin, T | Excluded | Insufficient progression velocity data |
| | Factors for glaucoma progression and the effect of treatment: the early manifest glaucoma trial | Leske, MC; Heijl, A; Hussein, M; Bengtsson, B; Hyman, L; Komaroff, E | Excluded | Insufficient progression velocity data |
| | Reduction of intraocular pressure and glaucoma progression: results from the Early Manifest Glaucoma Trial | Heijl, A; Leske, MC; Bengtsson, B; Hyman, L; Hussein, M | Excluded | Insufficient progression velocity data |
| | Can Treatment With Citicoline Eyedrops Reduce Progression in Glaucoma? The Results of a Randomized Placebo-controlled Clinical Trial | Rossetti, L; Iester, M; Tranchina, L; Ottobelli, L; Coco, G; Calcatelli, E; Ancona, C; Cirafici, P; Manni, G | Excluded | No IOP lowering therapy |
| | The United Kingdom Glaucoma Treatment Study: a multicenter, randomized, placebo-controlled clinical trial: design and methodology | Garway-Heath, DF; Lascaratos, G; Bunce, C; Crabb, DP; Russell, RA; Shah, A | Excluded | No results |
| | Disc hemorrhages and treatment in the early manifest glaucoma trial | Bengtsson, B; Leske, MC; Yang, Z; Heijl, A | Excluded | Did not measure glaucoma progression |
| | Long-term Impact of Immediate Versus Delayed Treatment of Early Glaucoma: results From the Early Manifest Glaucoma Trial | Heijl, A; Peters, D; Bengtsson, B | Excluded | Insufficient progression velocity data |
| | Intraocular pressure reduction with a fixed treatment protocol in the Early Manifest Glaucoma Trial | Heijl, A; Leske, MC; Hyman, L; Yang, Z; Bengtsson, B | Excluded | Insufficient progression velocity data |
| | Predictors of long-term progression in the early manifest glaucoma trial | Leske, MC; Heijl, A; Hyman, L; Bengtsson, B; Dong, L; Yang, Z | Excluded | Insufficient progression velocity data |
| | The singapore 5-Fluorouracil trabeculectomy study: effects on intraocular pressure control and disease progression at 3 years | Wong, TT; Khaw, PT; Aung, T; Foster, PJ; Htoon, HM; Oen, FT; Gazzard, G; Husain, R; Devereux, JG; Minassian, D; et al. | Excluded | No IOP lowering therapy |
| | Intraocular pressure reduction with topical medications and progression of normal-tension glaucoma: a 12-year mean follow-up study | Kim, M; Kim, DM; Park, KH; Kim, TW; Jeoung, JW; Kim, SH | Excluded | Insufficient progression velocity data |
| | The Association between Medication Adherence and Visual Field Progression in the Collaborative Initial Glaucoma Treatment Study | Newman-Casey, PA; Niziol, LM; Gillespie, BW; Janz, NK; Lichter, PR; Musch, DC | Excluded | Insufficient progression velocity data |
| | Predictors for visual field progression and the effects of treatment with dorzolamide 2% or brinzolamide 1% each added to timolol 0.5% in primary open-angle glaucoma | Martínez, A; Sanchez-Salorio, M | Excluded | Insufficient progression velocity data |
| | Fluctuation of intraocular pressure and glaucoma progression in the early manifest glaucoma trial | Bengtsson, B; Leske, MC; Hyman, L; Heijl, A | Excluded | Insufficient progression velocity data |
| | Personalized Prediction of Glaucoma Progression Under Different Target Intraocular Pressure Levels Using Filtered Forecasting Methods | Kazemian, P; Lavieri, MS; Van Oyen, MP; Andrews, C; Stein, JD | Excluded | Insufficient progression velocity data |
| | The Advanced Glaucoma Intervention Study (AGIS): 9. Comparison of glaucoma outcomes in black and white patients within treatment groups | AGIS Investigators | Excluded | Insufficient progression velocity data |
| | Visual field progression in the Collaborative Initial Glaucoma Treatment Study the impact of treatment and other baseline factors | Musch, DC; Gillespie, BW; Lichter, PR; Niziol, LM; Janz, NK | Excluded | Insufficient progression velocity data |
| | Structural and Functional Progression in the Early Manifest Glaucoma Trial | Öhnell, H; Heijl, A; Brenner, L; Anderson, H; Bengtsson, B | Excluded | Insufficient progression velocity data |
| | Visual Field Outcomes from the Multicenter, Randomized Controlled Laser in Glaucoma and Ocular Hypertension Trial (LiGHT) | Wright, DM; Konstantakopoulou, E; Montesano, G; Nathwani, N; Garg, A; Garway-Heath, D; Crabb, DP; Gazzard, G | Excluded | Insufficient progression velocity data |
| | Efficacy of Repeat Selective Laser Trabeculoplasty in Medication-Naive Open-Angle Glaucoma and Ocular Hypertension during the LiGHT Trial | Garg, A; Vickerstaff, V; Nathwani, N; Garway-Heath, D; Konstantakopoulou, E; Ambler, G; Bunce, C; Wormald, R; Barton, K; Gazzard, G | Excluded | Insufficient progression velocity data |
| | Oral Memantine for the Treatment of Glaucoma: design and Results of 2 Randomized, Placebo-Controlled, Phase 3 Studies | Weinreb, RN; Liebmann, JM; Cioffi, GA; Goldberg, I; Brandt, JD; Johnson, CA; Zangwill, LM; Schneider, S; Badger, H; Bejanian, M | Excluded | No IOP lowering therapy |
| | Measuring visual field progression in the Early Manifest Glaucoma Trial | Heijl, A; Leske, MC; Bengtsson, B; Hussein, M | Excluded | No results |
| | Methodological variations in estimating apparent progressive visual field loss in clinical trials of glaucoma treatment | Katz, J; Congdon, N; Friedman, DS | Excluded | No IOP lowering therapy |
| | Progression of retinal nerve fibre layer damage in betaxolol- and timolol-treated glaucoma patients | Vainio-Jylhä, E; Vuori, ML; Nummelin, K | Excluded | Insufficient progression velocity data |
| | Risk factors for progression of normal-tension glaucoma under β-blocker monotherapy | Araie, M; Shirato, S; Yamazaki, Y; Matsumoto, C; Kitazawa, Y; Ohashi, Y | Excluded | Insufficient progression velocity data |
| | Centrally acting cholinomimetics in the complex therapy of progressive glaucomatous optic neuropathy | Erichev, VP; Mazurova, YV | Excluded | No IOP lowering therapy |
| | Results of the European Glaucoma Prevention Study | Miglior, S; Zeyen, T; Pfeiffer, N; Cunha-Vaz, J; Torri, V; Adamsons, I | Excluded | Insufficient progression velocity data |
| | Efficacy of argon laser trabeculoplasty: 3-year preliminary results of a prospective placebo-controlled study | Ustundag, C; Diestelhorst, M | Excluded | Insufficient progression velocity data |
| | Preperimetric normal tension glaucoma study: long-term clinical course and effect of therapeutic lowering of intraocular pressure | Jeong, JH; Park, KH; Jeoung, JW; Kim, DM | Excluded | Insufficient progression velocity data |
| | Visual field loss in patients with normal-tension glaucoma under topical nipradilol or timolol: subgroup and subfield analyses of the nipradilol-timolol study | Araie, M; Shirato, S; Yamazaki, Y; Kitazawa, Y; Ohashi, Y | Excluded | Insufficient progression velocity data |
| | Effects of oral brovincamine on visual field damage in patients with normal-tension glaucoma with low-normal intraocular pressure | Koseki, N; Araie, M; Yamagami, J; Shirato, S; Yamamoto, S | Excluded | No IOP lowering therapy |
| | Cataract extraction in the collaborative initial glaucoma treatment study: incidence, risk factors, and the effect of cataract progression and extraction on clinical and quality-of-life outcomes | Musch, DC; Gillespie, BW; Niziol, LM; Janz, NK; Wren, PA; Rockwood, EJ; Lichter, PR | Excluded | Irrelevant outcomes |
| | The glaucoma intensive treatment study: interim results from an ongoing longitudinal randomized clinical trial | Bengtsson, B; Lindén, C; Heijl, A; Andersson-Geimer, S; Aspberg, J; Jóhannesson, G | Excluded | Insufficient progression velocity data |
| | Collaborative Initial Glaucoma Treatment Study: a summary of results to date | Feiner, L; Piltz-Seymour, JR | Excluded | Insufficient progression velocity data |
| | Two-Year Visual Field Outcomes of the Treatment of Advanced Glaucoma Study (TAGS) | Montesano, G; Ometto, G; King, A; Garway-Heath, DF; Crabb, DP | Excluded | Insufficient progression velocity data |
| | Natural history of normal-tension glaucoma | Anderson, DR; Drance, SM; Schulzer, M | Excluded | No intervention |
| | Projected impact of travoprost versus both timolol and latanoprost on visual field deficit progression and costs among black glaucoma subjects | Halpern, MT; Covert, DW; Robin, AL | Excluded | No intervention |
| | Effect of medically lowering intraocular pressure in glaucoma suspects with high myopia (GSHM study): study protocol for a randomized controlled trial | Lin, FB; Da Chen, S; Song, YH; Wang, W; Jin, L; Liu, BQ; Liu, YH; Chen, ML; Gao, K; Friedman, DS; et al. | Excluded | Irrelevant study population |
| | YAG laser peripheral iridotomy for the prevention of pigment dispersion glaucoma a prospective, randomized, controlled trial | Scott, A; Kotecha, A; Bunce, C; Balidis, M; Garway-Heath, DF; Miller, MH; Wormald, R | Excluded | Irrelevant study population |
| | The effectiveness of intraocular pressure reduction in the treatment of normal-tension glaucoma. Collaborative Normal-Tension Glaucoma Study Group | Collaborative Normal-Tension Glaucoma Study Group | Excluded | Insufficient progression velocity data |
| | Predictive factors for glaucomatous visual field progression in the Advanced Glaucoma Intervention Study | Nouri-Mahdavi, K; Hoffman, D; Coleman, AL; Liu, G; Li, G; Gaasterland, D; Caprioli, J | Excluded | Insufficient progression velocity data |
| | The Heidelberg retina tomograph Glaucoma Probability Score: reproducibility and measurement of progression | Strouthidis, NG; Demirel, S; Asaoka, R; Cossio-Zuniga, C; Garway-Heath, DF | Excluded | No intervention |
| | Machine learning classifiers-based prediction of normal-tension glaucoma progression in young myopic patients | Lee, J; Kim, YK; Jeoung, JW; Ha, A; Kim, YW; Park, KH | Excluded | No intervention |
| | The relationship between progression of visual field defects and retrobulbar circulation in patients with glaucoma | Yamazaki, Y; Drance, SM | Excluded | No intervention |
| | Effect of Inconsistent Optical Coherence Tomography Scan Location on Glaucoma Progression Analysis | Hwang, YH; Song, MY | Excluded | No intervention |
| | Canadian Glaucoma Study: 2. risk factors for the progression of open-angle glaucoma | Chauhan, BC; Mikelberg, FS; Balaszi, AG; LeBlanc, RP; Lesk, MR; Trope, GE | Excluded | Insufficient progression velocity data |
| | Association of Fellow Eye With Study Eye Disease Trajectories and Need for Fellow Eye Treatment in Collaborative Initial Glaucoma Treatment Study (CIGTS) Participants | Niziol, LM; Gillespie, BW; Musch, DC | Excluded | Insufficient progression velocity data |
| | Longitudinal and cross-sectional analyses of visual field progression in participants of the Ocular Hypertension Treatment Study | Artes, PH; Chauhan, BC; Keltner, JL; Cello, KE; Johnson, CA; Anderson, DR; Gordon, MO; Kass, MA | Excluded | Insufficient progression velocity data |
| | The rate of visual field change in the ocular hypertension treatment study | Demirel, S; De Moraes, CG; Gardiner, SK; Liebmann, JM; Cioffi, GA; Ritch, R; Gordon, MO; Kass, MA | Excluded | Irrelevant study population |
| | Five-year follow-up optic disc findings of the Collaborative Initial Glaucoma Treatment Study | Parrish, RK; Feuer, WJ; Schiffman, JC; Lichter, PR; Musch, DC | Excluded | Irrelevant outcomes |
| | Effect of chronic nitrate treatment on retinal vessel caliber in open-angle glaucoma | Grunwald, JE; DuPont, J; Dreyer, EB | Excluded | Irrelevant outcomes |
| | Early Manifest Glaucoma Trial update 2004 | Erb, C | Excluded | Insufficient progression velocity data |
| | Intraocular pressure control and long-term visual field loss in the Collaborative Initial Glaucoma Treatment Study | Musch, DC; Gillespie, BW; Niziol, LM; Lichter, PR; Varma, R | Excluded | Insufficient progression velocity data |
| | Combining optical coherence tomography with visual field data to rapidly detect disease progression in glaucoma: a diagnostic accuracy study | Garway-Heath, DF; Zhu, H; Cheng, Q; Morgan, K; Frost, C; Crabb, DP; Ho, TA; Agiomyrgiannakis, Y | Excluded | Irrelevant outcomes |
| | Effect of palmitoylethanolamide on visual field damage progression in normal tension glaucoma patients: results of an open-label six-month follow-up | Costagliola, C; Romano, MR; dell'Omo, R; Russo, A; Mastropasqua, R; Semeraro, F | Excluded | No IOP lowering therapy |
| | Collaborative normal tension glaucoma study | Anderson, DR | Excluded | No results |
| | Comparison of glaucomatous progression between untreated patients with normal-tension glaucoma and patients with therapeutically reduced intraocular pressures. Collaborative Normal-Tension Glaucoma Study Group | Collaborative Normal-Tension Glaucoma Study Group | Excluded | Insufficient progression velocity data |
| | Lack of visual field improvement after initiation of intraocular pressure reducing treatment in the early manifest glaucoma trial | Bengtsson, B; Heijl, A | Excluded | Insufficient progression velocity data |
| | Normal visual field test results following glaucomatous visual field end points in the Ocular Hypertension Treatment Study | Keltner, JL; Johnson, CA; Levine, RA; Fan, J; Cello, KE; Kass, MA; Gordon, MO | Excluded | Insufficient progression velocity data |
| | Selective laser trabeculoplasty following failed combined phacoemulsification cataract extraction and ab interno trabeculectomy | Töteberg-Harms, M; Rhee, DJ | Excluded | Irrelevant outcomes |
| | The Collaborative Initial Glaucoma Treatment Study: study design, methods, and baseline characteristics of enrolled patients | Musch, DC; Lichter, PR; Guire, KE; Standardi, CL | Excluded | No results |
| | The effect of brimonidine tartrate on retinal blood flow in patients with ocular hypertension | Carlsson, AM; Chauhan, BC; Lee, AA; Leblanc, RP | Excluded | No IOP lowering therapy |
| | The choice of drainage device in complicated glaucomas: comparing Ahmed and Baerveldt implants | Moschos, MM; Nitoda, E; Gouliopoulos, N; Androudi, S; Damaskos, C; Laios, K; Bagkli, E; Garmpis, N; Kitsos, G | Excluded | Irrelevant outcomes |
| | Treatment and vision-related quality of life in the early manifest glaucoma trial | Hyman, LG; Komaroff, E; Heijl, A; Bengtsson, B; Leske, MC | Excluded | Irrelevant outcomes |
| | Risk Factors for Visual Field Deterioration in the United Kingdom Glaucoma Treatment Study | Founti, P; Bunce, C; Khawaja, AP; Doré, CJ; Mohamed-Noriega, J; Garway-Heath, DF | Excluded | Insufficient progression velocity data |
| | A randomized trial of brimonidine versus timolol in preserving visual function: results from the Low-Pressure Glaucoma Treatment Study | Krupin, T; Liebmann, JM; Greenfield, DS; Ritch, R; Gardiner, S | Excluded | Insufficient progression velocity data |
| | Viscocanalostomy versus trabeculectomy in patients with bilateral high-tension glaucoma | Yarangümeli, A; Güreser, S; Köz, OG; Elhan, AH; Kural, G | Excluded | Irrelevant outcomes |
| | Phacoemulsification vs phacotrabeculectomy in chronic angle-closure glaucoma with cataract: complications | Tham, CC; Kwong, YY; Leung, DY; Lam, SW; Li, FC; Chiu, TY; Chan, JC; Lam, DS; Lai, JS | Excluded | Irrelevant study population |
| | The Advanced Glaucoma Intervention Study (AGIS): 4. Comparison of treatment outcomes within race. Seven-year results | AGIS Investigators | Excluded | Insufficient progression velocity data |
| | Investigating the neuroprotective effect of Copolymer-1 in acute primary angle closure - Interim report of a randomized placebo-controlled double-masked clinical trial | Fan, KR; Baskaran, M; Nongpiur, ME; Htoon, HM; de Leon, JMS; Perera, SA; Belkin, M; Aung, T | Excluded | No IOP lowering therapy |
| | Ocular Biometric Risk Factors for Progression of Primary Angle Closure Disease: the Zhongshan Angle Closure Prevention Trial | Xu, BY; Friedman, DS; Foster, PJ; Jiang, Y; Porporato, N; Pardeshi, AA; Munoz, B; Aung, T; He, M | Excluded | Irrelevant study population |
| | Visual field progression 8 years after trabeculectomy in Asian eyes: results from the Singapore 5-Fluorouracil Study | Ang, BCH; Seen, S; Kumaran, A; De Leon, JMS; Seah, SKL; Foster, PJ; Gazzard, G; Htoon, HM; Khaw, PT; Aung, T; et al. | Excluded | No IOP lowering therapy |
| | Intraocular pressure fluctuation a risk factor for visual field progression at low intraocular pressures in the advanced glaucoma intervention study | Caprioli, J; Coleman, AL | Excluded | Insufficient progression velocity data |
| | The influence of long-term retinal protective therapy on glaucoma progression according to structural and functional tests | Strakhov, VV; Egorov, EA; Erichev, VP; Yartsev, AV; Petrov, SY; Dorofeev, DA | Excluded | No IOP lowering therapy |
| | The Low-pressure Glaucoma Treatment Study (LoGTS) study design and baseline characteristics of enrolled patients | Krupin, T; Liebmann, JM; Greenfield, DS; Rosenberg, LF; Ritch, R; Yang, JW | Excluded | No results |
| | Two-year randomized, placebo-controlled study of black currant anthocyanins on visual field in glaucoma | Ohguro, H; Ohguro, I; Katai, M; Tanaka, S | Excluded | No IOP lowering therapy |
| | Effects of dorzolamide 2% added to timolol maleate 0.5% on intraocular pressure, retrobulbar blood flow, and the progression of visual field damage in patients with primary open-angle glaucoma: a single-center, 4-year, open-label study | Martínez, A; Sánchez, M | Excluded | Insufficient progression velocity data |
| | Natural history of intraocular pressure in the early manifest glaucoma trial: a 6-year follow-up | Hyman, L; Heijl, A; Leske, MC; Bengtsson, B; Yang, Z | Excluded | Insufficient progression velocity data |
| | Fourteen-Year Outcome of Angle-Closure Prevention with Laser Iridotomy in the Zhongshan Angle-Closure Prevention Study: extended Follow-up of a Randomized Controlled Trial | Yuan, Y; Wang, W; Xiong, R; Zhang, J; Li, C; Yang, S; Friedman, DS; Foster, PJ; He, M | Excluded | Irrelevant study population |
| | The rate of structural change: the confocal scanning laser ophthalmoscopy ancillary study to the ocular hypertension treatment study | Zangwill, LM; Jain, S; Dirkes, K; He, F; Medeiros, FA; Trick, GL; Brandt, JD; Cioffi, GA; Coleman, AL; Liebmann, JM; et al. | Excluded | Irrelevant outcomes |
| | Selective laser trabeculoplasty following failed combined phacoemulsification cataract extraction and excimer laser trabeculotomy can control intraocular pressure for a limited time | Krzyzanowska, I; Ziegler, J; Meier-Gibbons, F; Toteberg-Harms, M | Excluded | Irrelevant outcomes |
| | Comparative evaluation of diode laser trabeculoplasty vs frequency doubled Nd : YAG laser trabeculoplasty in primary open angle glaucoma | Agarwal, HC; Poovali, S; Sihota, R; Dada, T | Excluded | Irrelevant outcomes |
| | Travoprost versus latanoprost combinations in glaucoma: economic evaluation based on visual field deficit progression | Schmier, JK; Halpern, MT; Covert, DW; Robin, AL | Excluded | Insufficient progression velocity data |
| | Correlation between high-pass resolution perimetry and standard threshold perimetry in subjects with glaucoma and ocular hypertension | Iester, M; Capris, P; Altieri, M; Zingirian, M; Traverso, CE | Excluded | Irrelevant outcomes |
| | Laser in Glaucoma and Ocular Hypertension (LiGHT) Trial: six-Year Results of Primary Selective Laser Trabeculoplasty versus Eye Drops for the Treatment of Glaucoma and Ocular Hypertension | Gazzard, G; Konstantakopoulou, E; Garway-Heath, D; Adeleke, M; Vickerstaff, V; Ambler, G; Hunter, R; Bunce, C; Nathwani, N; Barton, K | Excluded | Insufficient progression velocity data |
| | Treatment of raised intraocular pressure and prevention of glaucoma | Wormald, R | Excluded | Irrelevant outcomes |
| | Evaluation of Visual Field and Imaging Outcomes for Glaucoma Clinical Trials (An American Ophthalomological Society Thesis) | Garway-Heath, DF; Quartilho, A; Prah, P; Crabb, DP; Cheng, Q; Zhu, H | Excluded | Insufficient progression velocity data |
| | The Advanced Glaucoma Intervention Study (AGIS): 7. The relationship between control of intraocular pressure and visual field deterioration.The AGIS Investigators | AGIS Investigators | Excluded | Insufficient progression velocity data |
| | Direct costs of glaucoma management following initiation of medical therapy. A simulation model based on an observational study of glaucoma treatment in Germany | Kobelt, G; Jönsson, L; Gerdtham, U; Krieglstein, GK | Excluded | Irrelevant outcomes |
| | Detection of glaucoma progression by perimetry and optic disc photography at different stages of the disease: results from the Early Manifest Glaucoma Trial | Öhnell, H; Heijl, A; Anderson, H; Bengtsson, B | Excluded | Insufficient progression velocity data |
| | Short wavelength automated perimetry, frequency doubling technology perimetry, and pattern electroretinography for prediction of progressive glaucomatous standard visual field defects | Bayer, AU; Erb, C | Excluded | No IOP lowering therapy |
| | Factors that predict the benefit of lowering intraocular pressure in normal tension glaucoma | Anderson, DR; Drance, SM; Schulzer, M | Excluded | Irrelevant outcomes |
| | Trabeculectomy with intraoperative mitomycin C versus 5-fluorouracil. Prospective randomized clinical trial | Singh, K; Mehta, K; Shaikh, NM; Tsai, JC; Moster, MR; Budenz, DL; Greenfield, DS; Chen, PP; Cohen, JS; Baerveldt, GS; et al. | Excluded | Irrelevant outcomes |
| | Five-Year Visual Field Outcomes of the HORIZON Trial | Montesano, G; Ometto, G; Ahmed, IIK; Ramulu, PY; Chang, DF; Crabb, DP; Gazzard, G | Excluded | Irrelevant outcomes |
| | The effects of postoperative corticosteroids on trabeculectomy and the clinical course of glaucoma: five-year follow-up study | Roth, SM; Spaeth, GL; Starita, RJ; Birbillis, EM; Steinmann, WC | Excluded | Irrelevant outcomes |
| | Body mass index in glaucoma | Gasser, P; Stümpfig, D; Schötzau, A; Ackermann-Liebrich, U; Flammer, J | Excluded | No IOP lowering therapy |
| | Primary Selective Laser Trabeculoplasty for Open-Angle Glaucoma and Ocular Hypertension: clinical Outcomes, Predictors of Success, and Safety from the Laser in Glaucoma and Ocular Hypertension Trial | Garg, A; Vickerstaff, V; Nathwani, N; Garway-Heath, D; Konstantakopoulou, E; Ambler, G; Bunce, C; Wormald, R; Barton, K; Gazzard, G | Excluded | Insufficient progression velocity data |
| | Latanoprost for open-angle glaucoma (UKGTS): a randomised, multicentre, placebo-controlled trial | Garway-Heath, DF; Crabb, DP; Bunce, C; Lascaratos, G; Amalfitano, F; Anand, N; Azuara-Blanco, A; Bourne, RR; Broadway, DC; Cunliffe, IA; et al. | Excluded | Insufficient progression velocity data |
| | Long-term effect of YAG laser iridotomy on corneal endothelium in primary angle closure suspects: a 72-month randomised controlled study | Liao, C; Zhang, J; Jiang, Y; Huang, S; Aung, T; Foster, PJ; Friedman, D; He, M | Excluded | Irrelevant study population |
| | The size of subconjunctival preparation does not influence the outcome of trabeculectomy with mitomycin C | Wasielica-Poslednik, J; Hoffmann, EM; Herzog, D; Aliyeva, S; Lamparter, J; Pfeiffer, N | Excluded | No IOP lowering therapy |
| | Laser peripheral iridotomy versus laser peripheral iridotomy plus laser peripheral iridoplasty in the treatment of multi-mechanism angle closure: study protocol for a randomized controlled trial | Chen, S; Lv, J; Fan, S; Zhang, H; Xie, L; Xu, L; Jiang, B; Yuan, H; Liang, Y; Li, S; et al. | Excluded | Irrelevant study population |
| | Early Manifest Glaucoma Trial: design and baseline data | Leske, MC; Heijl, A; Hyman, L; Bengtsson, B | Excluded | No results |
| | Safety and efficacy of using off-label bevacizumab versus mitomycin C to prevent bleb failure in a single-site phacotrabeculectomy by a randomized controlled clinical trial | Sengupta, S; Venkatesh, R; Ravindran, RD | Excluded | Irrelevant outcomes |
| | Predictive factors for open-angle glaucoma among patients with ocular hypertension in the European Glaucoma Prevention Study | Miglior, S; Pfeiffer, N; Torri, V; Zeyen, T; Cunha-Vaz, J; Adamsons, I | Excluded | Insufficient progression velocity data |
| | Quantifying the effect of intraocular pressure reduction on the occurrence of glaucoma | Peeters, A; Webers, CA; Prins, MH; Zeegers, MP; Hendrikse, F; Schouten, JS | Excluded | Irrelevant study population |
| | The Effect of an Educational Intervention on Adherence to Intraocular Pressure-Lowering Medications in a Large Cohort of Older Adults with Glaucoma | Fiscella, R; Caplan, E; Kamble, P; Bunniran, S; Uribe, C; Chandwani, H | Excluded | No IOP lowering therapy |
| | Natural history of open-angle glaucoma | Heijl, A; Bengtsson, B; Hyman, L; Leske, MC | Excluded | No intervention |
| | Evaluation of a Primary Open-Angle Glaucoma Prediction Model Using Long-term Intraocular Pressure Variability Data: a Secondary Analysis of 2 Randomized Clinical Trials | Gordon, MO; Gao, F; Huecker, JB; Miller, JP; Margolis, M; Kass, MA; Miglior, S; Torri, V | Excluded | No intervention |
| | OHTS 13 years later | Munoz-Negrete, FJ; Rebolleda, G; Ruiz-Casas, D | Excluded | Insufficient progression velocity data |
| | Effects of argon laser trabeculoplasty in the Early Manifest Glaucoma Trial | Heijl, A; Peters, D; Leske, MC; Bengtsson, B | Excluded | Insufficient progression velocity data |
| | A phase III study of subconjunctival human anti-transforming growth factor beta(2) monoclonal antibody (CAT-152) to prevent scarring after first-time trabeculectomy | Khaw, P; Grehn, F; Holló, G; Overton, B; Wilson, R; Vogel, R; Smith, Z | Excluded | No IOP lowering therapy |
| | Long-term intraocular pressure fluctuation and progressive visual field deterioration in patients with glaucoma and low intraocular pressures after a triple procedure | Hong, S; Seong, GJ; Hong, YJ | Excluded | Insufficient progression velocity data |
| | Effects of lens extraction versus laser peripheral iridotomy on anterior segment morphology in primary angle closure suspect | Yan, C; Han, Y; Yu, Y; Wang, W; Lyu, D; Tang, Y; Yao, K | Excluded | Irrelevant study population |
| | Canadian Glaucoma Study: 1. Study design, baseline characteristics, and preliminary analyses | Canadian Glaucoma Study Group 1 | Excluded | No results |
| | Results of the Collaborative Initial Glaucoma Treatment Study (CIGTS) | Wahl, J | Excluded | Insufficient progression velocity data |
| | Effect of IOP Lowering on Progressive HM | NCT05850936, | Excluded | Irrelevant study population |
| | Factors Associated with Worsening Proliferative Diabetic Retinopathy in Eyes Treated with Panretinal Photocoagulation or Ranibizumab | Bressler, SB; Beaulieu, WT; Glassman, AR; Gross, JG; Jampol, LM; Melia, M; Peters, MA; Rauser, ME | Excluded | Irrelevant study population |
| | Optic disc and visual field changes after trabeculectomy | Kotecha, A; Spratt, A; Bunce, C; Garway-Heath, DF; Khaw, PT; Viswanathan, A | Excluded | Insufficient progression velocity data |
| | Brimonidine vs ALTP in Progressing Human Glaucoma | NCT00466479, | Excluded | Insufficient progression velocity data |
| | Making a Correct Diagnosis of Glaucoma: data From the EMGT | Öhnell, H; Bengtsson, B; Heijl, A | Excluded | Insufficient progression velocity data |
| | Impact of segmentation errors and retinal blood vessels on retinal nerve fibre layer measurements using spectral-domain optical coherence tomography | Ye, C; Yu, M; Leung, CK | Excluded | No IOP lowering therapy |
| | Randomised controlled trial comparing the effect of brimonidine and timolol on visual field loss after acute primary angle closure | Aung, T; Oen, FT; Wong, HT; Chan, YH; Khoo, BK; Liu, YP; Ho, CL; See, J; Thean, LH; Viswanathan, AC; et al. | Excluded | Irrelevant study population |
| | Effect of trabeculectomy on lens opacities in an East Asian population | Husain, R; Aung, T; Gazzard, G; Foster, PJ; Devereux, JG; Chew, PT; Oen, FT; Khaw, PT; Seah, SK | Excluded | Irrelevant outcomes |
| | Diabetic eye disease: a primary care perspective | Frank, KJ; Dieckert, JP | Excluded | Irrelevant study population |
| | The Health Of Patients' Eyes (HOPE) Glaucoma study. The effectiveness of a 'glaucoma personal record' for newly diagnosed glaucoma patients: study protocol for a randomised controlled trial | Forbes, M; Fairlamb, H; Jonker, L | Excluded | No IOP lowering therapy |
| | Neodymium: YAG and argon laser iridotomy. Long-term follow-up in a prospective, randomized clinical trial | Del Priore, LV; Robin, AL; Pollack, IP | Excluded | Irrelevant study population |
| | A 1-year randomized study of the clinical and confocal effects of tafluprost and latanoprost in newly diagnosed glaucoma patients | Fogagnolo, P; Dipinto, A; Vanzulli, E; Maggiolo, E; De Cilla', S; Autelitano, A; Rossetti, L | Excluded | Insufficient progression velocity data |
| | Use of trabeculectomy with postoperative 5-fluorouracil in patients requiring extremely low intraocular pressure levels to limit further glaucoma progression | Wilson, RP; Steinmann, WC | Excluded | Insufficient progression velocity data |
| | Phacoemulsification versus Phacotrabeculectomy in Medically Controlled Primary Angle Closure Glaucoma with Cataract in an Indian Cohort: a randomized controlled trial | Senthil, S; Rao, HL; Choudhari, N; Garudadri, C | Excluded | Irrelevant study population |
| | Diurnal IOP fluctuation: not an independent risk factor for glaucomatous visual field loss in high-risk ocular hypertension | Bengtsson, B; Heijl, A | Excluded | No IOP lowering therapy |
| | Retinal nerve fiber layer atrophy is associated with visual field loss over time in glaucoma suspect and glaucomatous eyes | Sehi, M; Zhang, X; Greenfield, DS; Chung, Y; Wollstein, G; Francis, BA; Schuman, JS; Varma, R; Huang, D | Excluded | No IOP lowering therapy |
| | Selective laser trabeculoplasty versus drops for newly diagnosed ocular hypertension and glaucoma: the LiGHT RCT | Gazzard, G; Konstantakopoulou, E; Garway-Heath, D; Garg, A; Vickerstaff, V; Hunter, R; Ambler, G; Bunce, C; Wormald, R; Nathwani, N; et al. | Excluded | Insufficient progression velocity data |
| | Postoperative Complications in the Primary Tube Versus Trabeculectomy Study During 5 Years of Follow-up | Gedde, SJ; Feuer, WJ; Lim, KS; Barton, K; Goyal, S; Ahmed, II; Brandt, JD | Excluded | Irrelevant outcomes |
| | Comparing glaucomatous disc change using stereo disc viewing and the MatchedFlicker programme in glaucoma experts and trainees | Schaefer, JL; Meyer, AM; Rodgers, CD; Rosenberg, NC; Leoncavallo, AJ; Lukowski, ZL; Greer, AB; Martorana, GM; Zou, B; Shuster, JJ; et al. | Excluded | No IOP lowering therapy |
| | Latanoprost and brimonidine: therapeutic and physiologic assessment before and after oral nonsteroidal anti-inflammatory therapy | Sponsel, WE; Paris, G; Trigo, Y; Pena, M; Weber, A; Sanford, K; McKinnon, S | Excluded | Irrelevant outcomes |
| | Comparison of the effects of brimonidine 0.2% and timolol 0.5% on retinal nerve fiber layer thickness in ocular hypertensive patients: a prospective, unmasked study | Tsai, JC; Chang, HW | Excluded | Irrelevant outcomes |
| | Ocular hypertension: correlation of anterior chamber angle width and risk of progression to glaucoma | Wishart, PK; Batterbury, M | Excluded | No IOP lowering therapy |
| | Errors in the diagnosis of visual field progression in normal-tension glaucoma | Schulzer, M | Excluded | Irrelevant outcomes |
| | The Glaucoma Intensive Treatment Study (GITS), a randomized clinical trial: design, methodology and baseline data | Bengtsson, B; Heijl, A; Johannesson, G; Andersson-Geimer, S; Aspberg, J; Lindén, C | Excluded | No results |
| | Impact of Home Intraocular Pressure Telemonitoring on Intraocular Pressure Control and Glaucoma Progression | NCT05940623, | Excluded | No IOP lowering therapy |
| | The Efficacy of Citicoline in Eyedrops (OMK1) in Reducing the Progression of Glaucoma | NCT04020705, | Excluded | No IOP lowering therapy |
| | Treatment with beclomethasone dipropionate nasal aerosol in patients with perennial allergic rhinitis | Weinstein, SF; Schenkel, EJ; Bardelas, JA; Chylack Jr, LT; Tankelevich, A; Ding, Y; Tantry, SK | Excluded | No IOP lowering therapy |
| | A comparison of the effects of dorzolamide/timolol fixed combination versus latanoprost on intraocular pressure and pulsatile ocular blood flow in primary open-angle glaucoma patients | Janulevičienė, I; Harris, A; Kagemann, L; Siesky, B; McCranor, L | Excluded | Irrelevant outcomes |
| | Prevention of visual field defect progression with brovincamine in eyes with normal-tension glaucoma | Sawada, A; Kitazawa, Y; Yamamoto, T; Okabe, I; Ichien, K | Excluded | No IOP lowering therapy |
| | Ahmed glaucoma valve implantation with and without Ologen adjuvant in pediatric glaucoma | Elwehidy, AS; Bayoumi, NHL; Hagras, SM; Elshaer, S | Excluded | Irrelevant study population |
| | Nonpenetrating very deep sclerectomy with hyaluronic acid implant vs trabeculectomy--a 2-year follow-up | Leszczyński, R; Formińska-Kapuścik, M; Bubała-Stachowicz, B; Mrukwa-Kominek, E; Filipek, E; Pawlicki, K | Excluded | Irrelevant outcomes |
| | Intraoperative use of mitomycin C in glaucoma filtering surgery--indications, results and complications | Zaleska-Żmijewska, A; Szaflik, J | Excluded | Irrelevant outcomes |
| | Visual Field Outcomes in the Tube Versus Trabeculectomy Study | Swaminathan, SS; Jammal, AA; Kornmann, HL; Chen, PP; Feuer, WJ; Medeiros, FA; Gedde, SJ | Excluded | Insufficient progression velocity data |
| | Efficacy and safety of neuroprotection in patients with primary open-angle glaucoma | Malishevskaya, TN; Yusupov, AR; Shatskikh, SV; Antipina, NA; Klindyuk, TS; Bogdanova, DS | Excluded | No IOP lowering therapy |
| | Are Patient Self-Reported Outcome Measures Sensitive Enough to Be Used as End Points in Clinical Trials?: evidence from the United Kingdom Glaucoma Treatment Study | Jones, L; Garway-Heath, DF; Azuara-Blanco, A; Crabb, DP | Excluded | Irrelevant outcomes |
| | Nine-year follow-up of trabeculectomy with or without low-dosage mitomycin-c in primary open-angle glaucoma | Reibaldi, A; Uva, MG; Longo, A | Excluded | Irrelevant outcomes |
| | Hypotensive action of naturally occurring diterpenes: a therapeutic promise for the treatment of hypertension | Tirapelli, CR; Ambrosio, SR; De Oliveira, AM; Tostes, RC | Excluded | Irrelevant outcomes |
| | Efficacy and safety of Bujing Yishi tablet for glaucoma with controlled IOP: study protocol for a multi-centre randomized controlled trial | Liu, H; Li, X; Zhang, Z; Zeng, J; Dai, Y; Wang, C; Xie, Z; Cheng, L; Cui, L | Excluded | No IOP lowering therapy |
| | Randomised Clinical Trial for Postoperative Complications after Ex-PRESS Implantation versus Trabeculectomy with 2-Year Follow-Up | Arimura, S; Miyake, S; Iwasaki, K; Gozawa, M; Matsumura, T; Takamura, Y; Inatani, M | Excluded | Irrelevant outcomes |
| | Timolol treatment prevents or delays glaucomatous visual field loss in individuals with ocular hypertension: a five-year, randomized, double-masked, clinical trial | Kass, MA | Excluded | Insufficient progression velocity data |
| | Pneumatic trabeculoplasty vs latanoprost as adjunctive therapy to timolol in primary open-angle glaucoma or ocular hypertension | Uva, MG; Longo, A; Reibaldi, M | Excluded | Irrelevant outcomes |
| | Phacoemulsification versus combined phacotrabeculectomy in medically controlled chronic angle closure glaucoma with cataract | Tham, CC; Kwong, YY; Leung, DY; Lam, SW; Li, FC; Chiu, TY; Chan, JC; Chan, CH; Poon, AS; Yick, DW; et al. | Excluded | Irrelevant study population |
| | Supraciliary Micro-stent Implantation for Open-Angle Glaucoma Failing Topical Therapy: 1-Year Results of a Multicenter Study | García-Feijoo, J; Rau, M; Grisanti, S; Höh, H; Erb, C; Guguchkova, P; Ahmed, I; Grabner, G; Reitsamer, H; et al. | Excluded | Irrelevant outcomes |
| | The Singapore Asymptomatic Narrow Angles Laser Iridotomy Study: five-Year Results of a Randomized Controlled Trial | Baskaran, M; Kumar, RS; Friedman, DS; Lu, QS; Wong, HT; Chew, PTK; Lavanya, R; Narayanaswamy, A; Perera, SA; Foster, PJ; et al. | Excluded | Irrelevant study population |
| | A long-term dose-response study of mitomycin in glaucoma filtration surgery | Robin, AL; Ramakrishnan, R; Krishnadas, R; Smith, SD; Katz, JD; Selvaraj, S; Skuta, GL; Bhatnagar, R | Excluded | Irrelevant outcomes |
| | Treatment of rubeosis iridis with photodynamic therapy with verteporfin--A new therapeutic and prophylactic option for patients with the risk of neovascular glaucoma? | Müller, VA; Ruokonen, P; Schellenbeck, M; Hartmann, C; Tetz, M | Excluded | Irrelevant study population |
| | Phacoemulsification versus combined phacotrabeculectomy in medically uncontrolled chronic angle closure glaucoma with cataracts | Tham, CC; Kwong, YY; Leung, DY; Lam, SW; Li, FC; Chiu, TY; Chan, JC; Lam, DS; Lai, JS | Excluded | Irrelevant study population |
| | Therapeutic effect of dexamethasone implant in retinal vein occlusions resistant to anti-VEGF therapy | Wallsh, J; Sharareh, B; Gallemore, R | Excluded | Irrelevant study population |
| | Use of a Bayesian network to predict the nighttime intraocular pressure peak from daytime measurements | Nordmann, JP; Berdeaux, G | Excluded | No IOP lowering therapy |
| | Comparison of Ocular Pulse Amplitude-Lowering Effects of Tafluprost and Latanoprost by Dynamic Contour Tonometry | Park, SH; Yoo, SH; Ha, SJ | Excluded | Irrelevant outcomes |
| | Fluocinolone acetonide implant (Retisert) for noninfectious posterior uveitis: thirty-four-week results of a multicenter randomized clinical study | Jaffe, GJ; Martin, D; Callanan, D; Pearson, PA; Levy, B; Comstock, T | Excluded | Irrelevant study population |
| | Comparison of therapeutic effects of two CCBs on glaucoma and analysis of their possible mechanisms | Liang, T; Zhang, L; Gao, Y; Xiang, Y | Excluded | Irrelevant outcomes |
| | Viscocanalostomy versus trabeculectomy for primary open angle glaucoma: 4-year prospective randomized clinical trial | Gilmour, DF; Manners, TD; Devonport, H; Varga, Z; Solebo, AL; Miles, J | Excluded | Irrelevant outcomes |
| | Glaucoma management in developing countries: medical, laser, and surgical options for glaucoma management in countries with limited resources | Thomas, R; Sekhar, GC; Kumar, RS | Excluded | No IOP lowering therapy |
| | Medical glaucoma therapy | Jordan, JF; Dietlein, TS; Dinslage, S; Krieglstein, GK | Excluded | No IOP lowering therapy |
| | Influence of carteolol and timolol on IOP an visual fields in glaucoma: a multi-center, double-masked, prospective study | Flammer, J; Kitazawa, Y; Bonomi, L; Mills, B; Fsadni, M; Dorigo, MT; Shirato, S; Journel, B; Chavy, B; Chevallier, B | Excluded | Irrelevant outcomes |
| | Black currant anthocyanins normalized abnormal levels of serum concentrations of endothelin-1 in patients with glaucoma | Yoshida, K; Ohguro, I; Ohguro, H | Excluded | Insufficient progression velocity data |
| | Evaluation of retinal haemodynamics and retinal function after application of dorzolamide, timolol and latanoprost in newly diagnosed open-angle glaucoma patients | Arend, O; Harris, A; Wolter, P; Remky, A | Excluded | Irrelevant outcomes |
| | Ocular hypotensive effect of atenolol (Tenormin, I.C.I.). A new beta-adrenergic blocker | Elliot, MJ; Cullen, PM; Phillips, CI | Excluded | Irrelevant outcomes |
| | Preservative-free versus preserved latanoprost eye drops in patients with open-angle glaucoma or ocular hypertension | Aptel, F; Choudhry, R; Stalmans, I | Excluded | Insufficient progression velocity data |
| | Intraocular pressure fluctuations in response to the water-drinking provocative test in patients using latanoprost versus unoprostone | Susanna, R; Medeiros, FA; Vessani, RM; Giampani, J; Borges, AS; Jordão, ML | Excluded | No IOP lowering therapy |
| | Effect of naftidrofuryl (Praxilene) in primary open angle glaucoma: prospective double-blind study | Beati, D; Mermoud, A; Faggioni, R | Excluded | No IOP lowering therapy |
| | The ocular hypotensive effect of saffron extract in primary open angle glaucoma: a pilot study | Jabbarpoor Bonyadi, MH; Yazdani, S; Saadat, S | Excluded | Irrelevant outcomes |
| | Effects of travoprost 0.004% ophthalmic solution, six weeks after its laminated packaging had been removed, in primary open-angle glaucoma: a randomized, controlled, investigator-blinded study | Reis, R; dos Santos, LC; Vila, MP; Magacho, L | Excluded | Irrelevant outcomes |
| | Changes in intraocular pressure and ocular perfusion pressure after latanoprost 0.005% or brimonidine tartrate 0.2% in normal-tension glaucoma patients | Liu, CJ; Ko, YC; Cheng, CY; Chiu, AW; Chou, JC; Hsu, WM; Liu, JH | Excluded | Irrelevant outcomes |
| | Phase 3, Randomized, 20-Month Study of the Efficacy and Safety of Bimatoprost Implant in Patients with Open-Angle Glaucoma and Ocular Hypertension (ARTEMIS 2) | Bacharach, J; Tatham, A; Ferguson, G; Belalcázar, S; Thieme, H; Goodkin, ML; Chen, MY; Guo, Q; Liu, J; Robinson, MR; et al. | Excluded | Irrelevant outcomes |
| | Investigation of the Efficacy and Safety of 650 nm Low-Level Red Light for Myopia Control in Children: a Randomized Controlled Trial | Tian, L; Cao, K; Ma, D-L; Zhao, S-Q; Lu, L-X; Li, A; Chen, C-X; Ma, C-R; Ma, Z-F; Jie, Y | Excluded | Irrelevant study population |
| | GlaucoT Glaucoma Treatment Glasses in the Treatment of Primary Open-Angle Glaucoma | NCT05253534, | Excluded | No IOP lowering therapy |
| | A comparison between peripheral iridectomy with thermal sclerostomy and trabeculectomy: a controlled study | Spaeth, GL; Poryzees, E | Excluded | Irrelevant study population |
| | Effect of latanoprost 0.005% and brimonidine tartrate 0.2% on pulsatile ocular blood flow in normal tension glaucoma | Liu, CJ; Ko, YC; Cheng, CY; Chou, JC; Hsu, WM; Liu, JH | Excluded | Irrelevant outcomes |
| | Collaborative Initial Glaucoma Treatment Study (CIGTS) | NCT00000149, | Excluded | No results |
| | African descent and glaucoma evaluation study (ADAGES): racial differences in the risk of developing visual field damage vary by level of IOP | Zangwill, LM; Khachatryan, N; Bowd, C; Liebmann, JM; Girkin, CA; Weinreb, RN; Sample, PA; Hammel, N; Sharpsten, L; Medeiros, FA | Excluded | No IOP lowering therapy |
| | A Small Molecule Inhibitor of VE-PTP Activates Tie2 in Schlemm's Canal Increasing Outflow Facility and Reducing Intraocular Pressure | Li, G; Nottebaum, AF; Brigell, M; Navarro, ID; Ipe, U; Mishra, S; Gomez-Caraballo, M; Schmitt, H; Soldo, B; Pakola, S; et al. | Excluded | No IOP lowering therapy |
| | Primary argon laser trabeculoplasty vs pilocarpine. IV. Long-term effects on optic nerve head | Bergeå, B; Bodin, L; Svedbergh, B | Excluded | Irrelevant outcomes |
| | Improving adherence to glaucoma medication: a randomised controlled trial of a patient-centred intervention (The Norwich Adherence Glaucoma Study) | Cate, H; Bhattacharya, D; Clark, A; Fordham, R; Holland, R; Broadway, DC | Excluded | No IOP lowering therapy |
| | Argon laser trabeculoplasty in narrow angle glaucoma | Wishart, PK; Nagasubramanian, S; Hitchings, RA | Excluded | Irrelevant study population |
| | Study of Medical Treatment of Low-Pressure (Normal Tension) Glaucoma | NCT00317577, | Excluded | Insufficient progression velocity data |
| | Cost-utility analysis of trabecular micro-bypass stents (TBS) in patients with mild-to-moderate open-angle Glaucoma in Italy | Fea, AM; Cattel, F; Gandolfi, S; Buseghin, G; Furneri, G; Costagliola, C | Excluded | Irrelevant outcomes |
| | EXTERNAL SLT Treatment in Patients With Uncontrolled OPEN ANGLE GLAUCOMA | NCT01384149, | Excluded | Insufficient progression velocity data |
| | Clinical profile and early surgical complications in the Cornea Donor Study | Mannis, MJ; Holland, EJ; Beck, RW; Belin, MW; Goldberg, MA; Gal, RL; Kalajian, AD; Kenyon, KR; Kollman, C; Ruedy, KJ; et al. | Excluded | No IOP lowering therapy |
| | Low-dose and high-dose mitomycin trabeculectomy as an initial surgery in primary open-angle glaucoma | Kitazawa, Y; Suemori-Matsushita, H; Yamamoto, T; Kawase, K | Excluded | Insufficient progression velocity data |
| | Short-Term Intraocular Pressure Response to the Combined Effect of Transcutaneous Electrical Nerve Stimulation over Acupoint (Acu-TENS) and Yoga Ocular Exercise in Type 2 Diabetic Patients with Primary Open-Angle Glaucoma: a Randomized Controlled Trial | Ismail, AMA; El-Azeim, ASA | Excluded | Irrelevant outcomes |
| | A ten-year follow-up on a prospective, randomized trial of postoperative corticosteroids after trabeculectomy | Araujo, SV; Spaeth, GL; Roth, SM; Starita, RJ | Excluded | Irrelevant outcomes |
| | Mitomycin C-augmented Phacotrabeculectomy Versus Phacoemulsification in Primary Angle-closure Glaucoma: a Randomized Controlled Study | El Sayed, YM; Elhusseiny, AM; Albalkini, AS; El Sheikh, RH; Osman, MA | Excluded | Irrelevant study population |
| | Postoperative complications in the Tube Versus Trabeculectomy (TVT) study during five years of follow-up | Gedde, SJ; Herndon, LW; Brandt, JD; Budenz, DL; Feuer, WJ; Schiffman, JC | Excluded | Irrelevant outcomes |
| | Fear of blindness in the Collaborative Initial Glaucoma Treatment Study: patterns and correlates over time | Janz, NK; Wren, PA; Guire, KE; Musch, DC; Gillespie, BW; Lichter, PR | Excluded | Irrelevant outcomes |
| | A placebo-controlled 3-year study of a calcium blocker on visual field and ocular circulation in glaucoma with low-normal pressure | Koseki, N; Araie, M; Tomidokoro, A; Nagahara, M; Hasegawa, T; Tamaki, Y; Yamamoto, S | Excluded | No IOP lowering therapy |
| | A Protective Eye Shield Reduces Limbal Strain and Its Variability During Simulated Sleep in Adults With Glaucoma | Flatau, A; Solano, F; Jefferys, JL; Damion, C; Quigley, HA | Excluded | No IOP lowering therapy |
| | Comparison of Surgical Treatment Options for Primary Congenital and Developmental Glaucomas | NCT04647929, | Excluded | Irrelevant study population |
| | Topical timolol administration reduces the incidence of glaucomatous damage in ocular hypertensive individuals. A randomized, double-masked, long-term clinical trial | Kass, MA; Gordon, MO; Hoff, MR; Parkinson, JM; Kolker, AE; Hart, WM; Becker, B | Excluded | Irrelevant outcomes |
| | A 10-year follow-up to determine the effect of YAG laser iridotomy on the natural history of pigment dispersion syndrome: a randomized clinical trial | Gandolfi, SA; Ungaro, N; Tardini, MG; Ghirardini, S; Carta, A; Mora, P | Excluded | Irrelevant study population |
| | Baerveldt 350 vs Ahmed ClearPath 250 for the Treatment of Glaucoma | NCT04542616, | Excluded | Irrelevant outcomes |
| | Screening for primary open-angle glaucoma in the primary care setting: an update for the US Preventive Services Task Force | Fleming, C; Whitlock, EP; Beil, T; Smit, B; Harris, RP | Excluded | No IOP lowering therapy |
| | The blood-aqueous barrier changes after laser peripheral iridotomy or surgery peripheral iridectomy | Gao, XB; Zhang, XL; Chen, G; Huang, XK; Zhong, XJ; Lin, MK; Ge, J | Excluded | Irrelevant study population |
| | A cost-effectiveness analysis of iStent inject combined with phacoemulsification cataract surgery in patients with mild-to-moderate open-angle glaucoma in France | Nieland, K; Labbe, A; Schweitzer, C; Gicquel, G; Kleintjens, J; Ostawal, A; Treur, M; Falvey, H | Excluded | Irrelevant outcomes |
| | A long-term clinical trial of timolol therapy versus no treatment in the management of glaucoma suspects | Epstein, DL; Krug, JH; Hertzmark, E; Remis, LL; Edelstein, DJ | Excluded | Irrelevant study population |
| | A Trial of China Laser and Surgery Study Glaucoma in Rural China | NCT02955849, | Excluded | Irrelevant outcomes |
| | Meditation enhances brain oxygenation, upregulates BDNF and improves quality of life in patients with primary open angle glaucoma: a randomized controlled trial | Gagrani, M; Faiq, MA; Sidhu, T; Dada, R; Yadav, RK; Sihota, R; Kochhar, KP; Verma, R; Dada, T | Excluded | No IOP lowering therapy |
| | Natural history and clinical management of central retinal vein occlusion. The Central Vein Occlusion Study Group | The Central Vein Occlusion Study Group | Excluded | Irrelevant study population |
| | Early Manifest Glaucoma Trial (EMGT) | NCT00000132, | Excluded | No results |
| | Three-Year Outcomes of a Schlemm Canal Microstent (Hydrus Microstent) with Concomitant Phacoemulsification in Open-Angle Glaucoma | Salimi, A; Kassem, R; Santhakumaran, S; Harasymowycz, P | Excluded | Insufficient progression velocity data |
| | Effect of IOP Lowering in Glaucoma Suspects With HM | NCT04296916, | Excluded | Irrelevant study population |
| | The Glaucoma Nicotinamide Trial | NCT05275738, | Excluded | No IOP lowering therapy |
| | Glaucoma Biomarkers | NCT01677507, | Excluded | No intervention |
| | Ginkgo Biloba and Ocular Blood Flow in Primary Open-angle Glaucoma | NCT02376114, | Excluded | No IOP lowering therapy |
| | The Canadian Glaucoma Study | NCT00262626, | Excluded | No results |
| | OCT Signal Enhancement with Deep Learning | Lazaridis, G; Lorenzi, M; Mohamed-Noriega, J; Aguilar-Munoa, S; Suzuki, K; Nomoto, H; Ourselin, S; Garway-Heath, DF | Excluded | No intervention |
| | GTS400 Stent Implantation in Conjunction With Cataract Surgery in Subjects With Open-angle Glaucoma | NCT01052558, | Excluded | Irrelevant outcomes |
| | Lucentis for New Onset Neovascular Glaucoma | NCT00727038, | Excluded | Irrelevant study population |
| | Study of Neurotidine® Intake on Life Quality of Patients With Glaucoma | NCT04046809, | Excluded | No IOP lowering therapy |
| | Sensing Contact Lens-based Device for 24-hour Monitoring of Intraocular Pressure | NCT03689088, | Excluded | No IOP lowering therapy |
| | Effect of Metformin on Visual Function in Patients With Glaucoma | NCT04155164, | Excluded | No IOP lowering therapy |
| | Applying the recent clinical trials on primary open angle glaucoma: the developing world perspective | Thomas, R; Kumar, RS; Chandrasekhar, G; Parikh, R | Excluded | No intervention |
| | A Study to Assess an Automated Laser Device for Direct Selective Laser Trabeculoplasty | NCT03750201, | Excluded | Irrelevant outcomes |
| | Some clinical implications of the collaborative normal tension glaucoma study | Drance, SM | Excluded | Irrelevant outcomes |
| | Evaluating the Effects of an Ophthalmic Solution of Coenzyme Q10 and Vitamin E in Open-Angle Glaucoma Patients: a Study Protocol | Quaranta, L; Riva, I; Biagioli, E; Rulli, E; Poli, D; Legramandi, L | Excluded | No IOP lowering therapy |
| | Nicotinamide in Glaucoma | NCT05405868, | Excluded | No IOP lowering therapy |
| | Exercise Intervention in Primary Open-angle Glaucoma | NCT05704777, | Excluded | No IOP lowering therapy |
| | Study on Incentives for Glaucoma Medications Adherence | NCT02271269, | Excluded | No IOP lowering therapy |
| | The Bristol Shared Care Glaucoma Study: study design | Spencer, IC; Spry, PG; Gray, SF; Baker, IA; Menage, MJ; Easty, DL; Sparrow, JM | Excluded | No results |
| | Antisecretory Factor (AF) Effects on Intraocular-pressure | NCT02731118, | Excluded | Irrelevant outcomes |
| | Efficacy of Citicoline Eye Drops 2% on Visual Field Preservation in Patients With Open Angle Glaucoma | NCT05710198, | Excluded | No IOP lowering therapy |
| | Normal Tension and Chronic Open Angle Glaucoma and Cerebrospinal Fluid Composition | NCT00306657, | Excluded | No results available |
| | Stop Retinal Ganglion Cell Dysfunction Study | NCT02390284, | Excluded | No IOP lowering therapy |
| | Twenty-four hour intraocular pressure monitoring with the SENSIMED Triggerfish contact lens: effect of body posture during sleep | Beltran-Agulló, L; Buys, YM; Jahan, F; Shapiro, CM; Flanagan, JG; Cheng, J; Trope, GE | Excluded | No IOP lowering therapy |
| | Multicenter Study Assessing the Efficacy and Safety of DE-126 Ophthalmic Solution 0.002% Compared With Timolol Maleate Ophthalmic Solution 0.5% in Subjects With Primary Open Angle Glaucoma or Ocular Hypertension | NCT04742283, | Excluded | Irrelevant outcomes |
| | Metformin as a Neuroprotective Therapy for Glaucoma - A Randomized Controlled Trial | NCT05426044, | Excluded | No IOP lowering therapy |
| | The Laser in Pseudoexfoliation (LIP) Study | NCT01704989, | Excluded | Irrelevant study population |
| | Changes in the retrobulbar hemodynamic parameters after decreasing the elevated intraocular pressure in primary open-angle glaucoma patients | Marjanović, I; Martinez, A; Marjanović, M; Milić, N; Kontić, D; Hentova-Senćanić, P; Marković, V; Bozić, M | Excluded | Irrelevant outcomes |
| | The rise of intraocular pressure in a caffeine test versus the water drinking test in patients with glaucoma | Tran, T; Niyadurupola, N; O'Connor, J; Ang, GS; Crowston, J; Nguyen, D | Excluded | No IOP lowering therapy |
| | Comparison of Efficacy and Safety of Mitomycin and Aflibercept Used to Support Primary Trabeculectomy | NCT03766425, | Excluded | Irrelevant outcomes |
| | Circadian Rhythms of Aqueous Humor Dynamics in Humans | NCT00572936, | Excluded | No IOP lowering therapy |
| | Glaucoma Eye Drop Instillation: impact of Education | NCT01416415, | Excluded | No IOP lowering therapy |
| | A Comparison of the Effect of Dorzolamide and Timolol on Optic Disk Blood Flow in Patients With Open Angle Glaucoma | NCT00991822, | Excluded | Irrelevant outcomes |
| | A comparative study of small incision trabeculectomy avoiding tenon's capsule with conventional trabeculectomy | Das, JC; Sharma, P; Chaudhuri, Z; Bhomaj, S | Excluded | Irrelevant outcomes |
| | Shared Cared for Stable Glaucoma Patients: economic Benefits and Patient-centered Outcomes of a Feasibility Trial | Goh, D; de Korne, DF; Ho, H; Mathur, R; Chakraborty, B; Van Hai, N; Wai, C; Perera, S; Aung, T; Wong, TY; et al. | Excluded | Irrelevant outcomes |
| | Correlation between retinal nerve fiber layer thickness and IOP variation in glaucoma suspects and patients with primary open-angle glaucoma | Cronemberger, S; Veloso, AW; Veiga, C; Scarpelli, G; Sasso, YC; Merola, RV | Excluded | No IOP lowering therapy |
| | Cost-effectiveness analysis of standalone trabecular micro-bypass stents in patients with mild-to-moderate open-angle glaucoma in Canada | Patel, V; Ahmed, I; Podbielski, D; Falvey, H; Murray, J; Goeree, R | Excluded | Irrelevant outcomes |
| | Relationship of systemic blood pressure with ocular perfusion pressure and intraocular pressure of glaucoma patients in telemedical home monitoring | Jürgens, C; Grossjohann, R; Tost, FH | Excluded | No IOP lowering therapy |
| | Ten-Year Clinical Outcomes of Acute Primary Angle Closure Randomized to Receive Early Phacoemulsification Versus Laser Peripheral Iridotomy | Chan, PP; Tang, FY; Leung, DY; Lam, TC; Baig, N; Tham, CC | Excluded | Irrelevant study population |
| | Rise of intraocular pressure in a caffeine test versus the water drinking test in patients with glaucoma | Tran, T; Niyadurupola, N; O'Connor, J; Ang, GS; Crowston, J; Nguyen, D | Excluded | No IOP lowering therapy |
| | Goldmann applanation tonometry and dynamic contour tonometry are not correlated with central corneal thickness in primary open angle glaucoma | Regev, G; Harris, A; Siesky, B; Shoshani, Y; Egan, P; Moss, A; Zalish, M; WuDunn, D; Ehrlich, R | Excluded | No IOP lowering therapy |
| | Post-operative inflammation following deep sclerectomy with collagen implant versus standard trabeculectomy | Chiou, AG; Mermoud, A; Jewelewicz, DA | Excluded | No IOP lowering therapy |
| | Early diagnostic parameters of glaucoma in high myopes | Mohammad Noureldine, A; Hashem Fouad, P; Magdy Ahmed, H; Mahmoud Khafagy, M | Excluded | No intervention |
| | Advanced Imaging for Glaucoma Study | NCT01314326, | Excluded | No results |
| | Phase 1b Randomized Controlled Study of Short Course Topical Recombinant Human Nerve Growth Factor (rhNGF) for Neuroenhancement in Glaucoma: safety, Tolerability, and Efficacy Measure Outcomes | Beykin, G; Stell, L; Halim, MS; Nuñez, M; Popova, L; Nguyen, BT; Groth, SL; Dennis, A; Li, Z; Atkins, M; et al. | Excluded | No IOP lowering therapy |
| | Non-inferiority of PRO-122 Ophthalmic Solution vs KRYTANTEK Ofteno® in Glaucoma or Ocular Hypertension (CONFORTK) | NCT03257813, | Excluded | No IOP lowering therapy |
| | Non-inferiority of PRO-067 Ophthalmic Solution vs GAAP Ofteno® in Glaucoma or Ocular Hypertension | NCT02801617, | Excluded | No IOP lowering therapy |
| | Will Listening to Music Make it Easier to Take a Visual Field Test? | NCT01027039, | Excluded | No IOP lowering therapy |
| | Subconjunctival Versus Direct Mitomycin C in Trabeculectomy | NCT04352660, | Excluded | No IOP lowering therapy |
| | Combined Phacotube vs Phacotrabeculectomy: a Randomized Controlled Trial | NCT00273221, | Excluded | Irrelevant outcomes |
| | Relationship between visual field testing and scanning laser polarimetry in patients with a large cup-to-disk ratio | Tannenbaum, DP; Zangwill, LM; Bowd, C; Sample, PA; Weinreb, RN | Excluded | No IOP lowering therapy |
| | Blue-on-yellow visual field and retinal nerve fiber layer in ocular hypertension and glaucoma | Teesalu, P; Airaksinen, PJ; Tuulonen, A | Excluded | Irrelevant study population |
| | Bimatoprost Implant Biodegradation in the Phase 3, Randomized, 20-Month ARTEMIS Studies | Weinreb, RN; Bacharach, J; Brubaker, JW; Medeiros, FA; Bejanian, M; Bernstein, P; Robinson, MR | Excluded | Irrelevant outcomes |
| | The Diagnostic Ability of Ganglion Cell Complex Thickness-to-Total Retinal Thickness Ratio in Glaucoma in a Caucasian Population | Sarigul Sezenoz, A; Gur Gungor, S; Akman, A; Ozturk, C; Cezairlioglu, S; Aksoy, M; Colak, M | Excluded | No IOP lowering therapy |
| | Corneal epithelium and limbal region alterations due to glaucoma medications evaluated by anterior segment optic coherence tomography: a case-control study | Güçlü, H; Çınar, AK; Çınar, AC; Akaray, İ; Şambel Aykutlu, M; Sakallıoğlu, AK; Gürlü, V | Excluded | Irrelevant study population |
| | Efficacy of Glaucoma Drop Aids in Medication Compliance in the Patient Population at Boston Medical Center | NCT02867995, | Excluded | Irrelevant outcomes |
| | Primary trabeculectomy versus primary glaucoma eye drops for newly diagnosed advanced glaucoma: TAGS RCT | King, AJ; Fernie, G; Hudson, J; Kernohan, A; Azuara-Blanco, A; Burr, J; Homer, T; Shabaninejad, H; Sparrow, JM; Garway-Heath, D; et al. | Excluded | Insufficient progression velocity data |
| | Early trabeculectomy versus conventional management in primary open angle glaucoma | Jay, JL; Murray, SB | Excluded | Insufficient progression velocity data |
| | Completed 28-Day and ongoing 12-month safety and efficacy evaluation of env515 (travoprost) intracameral implant in phase 2 study | Walters, TR; Navratil, T; Pittman, S; Conley, V; Trevino, L; Silverstein, BE; Depenbusch, M; Mansberger, SL; Schiffman, R | Excluded | Irrelevant outcomes |
| | Timing of Glaucoma Drainage Device With Boston KPro Surgery | NCT02084745, | Excluded | Irrelevant outcomes |
| | Endothelial Cell Loss in Pseudophakic Patients Receiving a Paul Glaucoma Drainage Device With Its Tube Inserted in the Anterior Versus Posterior Chamber: a Randomized Controlled Trial | NCT05500469, | Excluded | Irrelevant study population |
| | Impact of patient access to Internet health records on glaucoma medication: randomized controlled trial | Kashiwagi, K; Tsukahara, S | Excluded | No IOP lowering therapy |
| | Intraocular Pressure Reduction Efficacy of Rhopressa and Lumigan in Normal Tension Glaucoma | NCT04981886, | Excluded | Irrelevant outcomes |
| | European Nutrition in Glaucoma Management Trial | NCT04460365, | Excluded | No IOP lowering therapy |
| | Kahook Dual Blade Ab-interno Trabeculotomy Versus ab Externo Viscotrabeculotomy in Primary Congenital Glaucoma | NCT05115708, | Excluded | Irrelevant study population |
| | Efficacy and Safety of ab Interno Trabeculectomy With the Kahook Dual Blade | NCT04202562, | Excluded | Irrelevant outcomes |
| | Panretinal photocoagulation and photodynamic therapy for anterior segment neovascularization secondary to ischemic central retinal vein occlusion | Parodi, MB; Friberg, TR; Pedio, M; Fiotti, N; Di Stefano, G; Ravalico, G | Excluded | Irrelevant study population |
| | Impact of patient-held record on knowledge at 1-year follow-up for glaucoma patients: single-center randomized controlled trial | Forbes, M; Fairlamb, H; Jonker, L | Excluded | No IOP lowering therapy |
| | Subconjunctival Bevacizumab to Prevent Bleb Failure After Glaucoma Filtration Surgery | NCT00468429, | Excluded | Irrelevant outcomes |
| | Decorin in Sub Scleral Trabeculectomy | NCT03924544, | Excluded | No IOP lowering therapy |
| | AGV Implantation With Ologen in Pediatric Glaucomas | NCT05014386, | Excluded | Irrelevant study population |
| | Inhaled Corticosteroids: effect on Intraocular Pressure in Patients With Controlled Glaucoma | NCT02338362, | Excluded | Irrelevant outcomes |
| | Study of Ocular Blood Flow in Patients With Glaucoma and/or Obstructive Sleep Apnea Syndrome (OSAS) | NCT00874913, | Excluded | No IOP lowering therapy |
| | Comparing the Effectiveness of Phacoemulsification + ECP Laser and Phacoemulsification Alone for Glaucoma Patients | NCT04295122, | Excluded | Irrelevant outcomes |
| | Visco-Circumferential-Suture-Trabeculotomy Versus Rigid Probe Viscotrabeculotomy in Primary Open Angle Glaucoma | NCT05666440, | Excluded | Irrelevant outcomes |
| | Comparing Phaco/IOL Versus Phaco/IOL + Goniosynechialysis in Subjects With PACG | NCT02376725, | Excluded | Irrelevant study population |
| | Malay Glaucoma Eye Study II Navigation, Mobility and Reading Ability in Primary Glaucoma | NCT04736264, | Excluded | No IOP lowering therapy |
| | Citicoline oral solution in glaucoma: is there a role in slowing disease progression? | Ottobelli, L.; Manni, G. L.; Centofanti, M.; Iester, M.; Allevena, F.; Rossetti, L. | Excluded | No IOP lowering therapy |
| | More Accurate Modeling of Visual Field Progression in Glaucoma: ANSWERS. | Zhu, Haogang; Crabb, David P.; Ho, Tuan; Garway-Heath, David F. | Excluded | No intervention |
| | Comparison of the effects of bimatoprost and a fixed combination of latanoprost and timolol on 24-hour blood and ocular perfusion pressures: the results of a randomized trial. | Rossetti, Luca; Sacchi, Matteo; Karabatsas, Costas H.; Topouzis, Fotis; Vetrugno, Michele; Centofanti, Marco; Boehm, Andreas; Vorwerk, Christian; Goldblum, David; Fogagnolo, Paolo | Excluded | Irrelevant outcomes |
| | Surgical complications in the Tube Versus Trabeculectomy Study during the first year of follow-up. | Gedde, Steven J.; Herndon, Leon W.; Brandt, James D.; Budenz, Donald L.; Feuer, William J.; Schiffman, Joyce C. | Excluded | No IOP lowering therapy |
| | Cataract progression after Nd:YAG laser iridotomy in primary angle-closure suspect eyes. | Chang, Dolly Shuo-Teh; Jiang, Yuzhen; Kim, Julia Anne; Huang, Shengsong; Munoz, Beatriz; Aung, Tin; He, Mingguang; Foster, Paul J.; Friedman, David | Excluded | Irrelevant study population |
| | Phacoemulsification vs phacotrabeculectomy in chronic angle-closure glaucoma with cataract: complications [corrected]. | Tham, Clement C. Y.; Kwong, Yolanda Y. Y.; Leung, Dexter Y. L.; Lam, Sze Wing; Li, Felix C. H.; Chiu, Thomas Y. H.; Chan, Jonathan C. H.; Lam, Dennis S. C.; Lai, Jimmy S. M. | Excluded | Irrelevant study population |
| | The effect of brimonidine tartrate on retinal blood flow in patients with ocular hypertension [corrected]. | Carlsson, A. M.; Chauhan, B. C.; Lee, A. A.; Leblanc, R. P. | Excluded | No IOP lowering therapy |
| | Using perimetric data to estimate ganglion cell loss for detecting progression of glaucoma: a comparison of models. | Price, Derek A.; Swanson, William H.; Horner, Douglas G. | Excluded | No intervention |
| | Twenty-four-hour control with latanoprost-timolol-fixed combination therapy vs latanoprost therapy. | Konstas, Anastasios G. P.; Boboridis, Kostantinos; Tzetzi, Despina; Kallinderis, Kostantinos; Jenkins, Jessica N.; Stewart, William C. | Excluded | Irrelevant outcomes |
| | A comparison of measures used to describe adherence to glaucoma medication in a randomised controlled trial. | Cate, Heidi; Bhattacharya, Debi; Clark, Allan; Holland, Richard; Broadway, David C. | Excluded | No intervention |
| | Melatonin mitigates disrupted circadian rhythms, lowers intraocular pressure, and improves retinal ganglion cells function in glaucoma. | Gubin D; Neroev V; Malishevskaya T; Cornelissen G; Astakhov SY; Kolomeichuk S; Yuzhakova N; Kabitskaya Y; Weinert D | Excluded | No IOP lowering therapy |
| | Intraocular Pressure Rise in Subjects with and without Glaucoma during Four Common Yoga Positions. | Jasien JV; Jonas JB; de Moraes CG; Ritch R | Excluded | No IOP lowering therapy |
| | Mean intraocular pressure and progression based on corneal thickness in patients with ocular hypertension. | Konstas AG; Irkec MT; Teus MA; Cvenkel B; Astakhov YS; Sharpe ED; Hollo G; Mylopoulos N; Bozkurt B; Pizzamiglio C; Potyomkin VV; Alemu AM; Nasser QJ; Stewart JA; Stewart WC | Excluded | No intervention |
| | Ibopamine challenge testing differentiates glaucoma suspect, stable glaucoma and progressive glaucoma cases. | Landers J; Ullrich K; Craig JE | Excluded | No IOP lowering therapy |
| | Simvastatin and disease stabilization in normal tension glaucoma: a cohort study. | Leung DY; Li FC; Kwong YY; Tham CC; Chi SC; Lam DS | Excluded | No IOP lowering therapy |
| | Association between Intraocular Pressure and Rates of Retinal Nerve Fiber Layer Loss Measured by Optical Coherence Tomography. | Diniz-Filho A; Abe RY; Zangwill LM; Gracitelli CP; Weinreb RN; Girkin CA; Liebmann JM; Medeiros FA | Excluded | No intervention |
| | Comparison of fluctuations of intraocular pressure before and after selective laser trabeculoplasty in normal-tension glaucoma patients. | Tojo N; Oka M; Miyakoshi A; Ozaki H; Hayashi A | Excluded | Irrelevant outcomes |
| | SHORT TERM EVALUATION OF PERIMETRIC PROGRESSION IN PATIENTS WITH OPEN ANGLE GLAUCOMA AND DIABETES. | Pantalon A; Feraru C; Chiseliţa D | Excluded | No IOP lowering therapy |
| | [Preservative-free glaucoma treatment : Selection of the correct treatment in 1 min]. | Pfennigsdorf S; Eschstruth P | Excluded | No intervention |
| | Comparative proteomic study in serum of patients with primary open-angle glaucoma and pseudoexfoliation glaucoma. | González-Iglesias H; Álvarez L; García M; Escribano J; Rodríguez-Calvo PP; Fernández-Vega L; Coca-Prados M | Excluded | No intervention |
| | Factors associated with long-term progression or stability in primary open-angle glaucoma. | Stewart WC; Kolker AE; Sharpe ED; Day DG; Holmes KT; Leech JN; Johnson M; Cantrell JB | Excluded | No intervention |
| | Series length used during trend analysis affects sensitivity to changes in progression rate in the ocular hypertension treatment study. | Gardiner SK; Demirel S; De Moraes CG; Liebmann JM; Cioffi GA; Ritch R; Gordon MO; Kass MA | Excluded | No intervention |
| | High intensity focused ultrasound as first line treatment in patients with chronic angle closure glaucoma at risk for malignant glaucoma. | Graber M; Khoueir Z; Beauchet A; Benhatchi N; Hammoud S; Lachkar Y | Excluded | Irrelevant study population |
| | 24-hour intraocular pressure control with maximum medical therapy compared with surgery in patients with advanced open-angle glaucoma. | Konstas AG; Topouzis F; Leliopoulou O; Pappas T; Georgiadis N; Jenkins JN; Stewart WC | Excluded | Irrelevant outcomes |
| | Comparison of the hypotensor effect between latanoprost versus selective laser trabeculoplasty obtained with the water drinking test. | Germano RAS; Hatanaka M; Garcia AS; Germano FAS; Germano CS; Cid FB; Germano JE | Excluded | Irrelevant outcomes |
| | Prospective evaluation of optic nerve head by confocal scanning laser ophthalmoscopy after intraocular pressure control in adult glaucoma. | Rao A; Sihota R; Srinivasan G; Gupta V; Gupta A; Sharma A | Excluded | Irrelevant outcomes |
| | Progress in glaucoma treatment research: a nonrandomized prospective study of 102 patients with advanced refractory glaucoma treated by Leksell gamma knife irradiation. | Vladyka V; Liscák R; Simonová G; Pilbauer J; Hejduková I; Novácek L | Excluded | Irrelevant outcomes |
| | A method to measure and predict rates of regional visual field decay in glaucoma. | Caprioli J; Mock D; Bitrian E; Afifi AA; Yu F; Nouri-Mahdavi K; Coleman AL | Excluded | No intervention |
| | Ocular response analyser to assess hysteresis and corneal resistance factor in low tension, open angle glaucoma and ocular hypertension. | Shah S; Laiquzzaman M; Mantry S; Cunliffe I | Excluded | No intervention |
| | The SECURE study: long-term safety of ranibizumab 0.5 mg in neovascular age-related macular degeneration. | Silva R; Axer-Siegel R; Eldem B; Guymer R; Kirchhof B; Papp A; Seres A; Gekkieva M; Nieweg A; Pilz S | Excluded | Irrelevant study population |
| | Multicenter, open-label evaluation of hyperemia associated with use of bimatoprost in adults with open-angle glaucoma or ocular hypertension. | Abelson MB; Mroz M; Rosner SA; Dirks MS; Hirabayashi D | Excluded | Irrelevant outcomes |
| | Increased corneal thickness in patients with ocular hypertension. | Herman DC; Hodge DO; Bourne WM | Excluded | No intervention |
| | Glaucomatous vertical vessel density asymmetry of the temporal raphe detected with optical coherence tomography angiography. | Yoshikawa Y; Shoji T; Kanno J; Ibuki H; Weinreb RN; Araie M; Shinoda K | Excluded | No intervention |
| | Efficacy and safety of bimatoprost in patients with uncontrolled glaucoma as alternative to filtration surgery. | Vetrugno M; Sborgia C; Balestrazzi E; Bianchi C; Caporossi A; Nardi M; Rapisarda A; Tassinari G; Zeppa L | Excluded | Insufficient progression velocity data |
| | Evaluation of offset of conjunctival hyperemia induced by a Rho-kinase inhibitor; 0.4% Ripasudil ophthalmic solution clinical trial. | Sakamoto E; Ishida W; Sumi T; Kishimoto T; Tada K; Fukuda K; Yoneda T; Kuroiwa H; Terao E; Fujisawa Y; Nakakura S; Jian K; Okumichi H; Kiuchi Y; Fukushima A | Excluded | Irrelevant outcomes |
| | The influence of topical prostaglandin analogues in inflammation after selective laser trabeculoplasty treatment. | Ayala M; Chen E | Excluded | Irrelevant outcomes |
| | Surgical outcomes of minimally invasive vitrectomy surgery in Eales' disease. | Khanduja S; Gupta S; Sinha S; Venkatesh P; Vohra R; Garg S | Excluded | No intervention |
| | Mitomycin-C in combined or two-stage procedure trabeculectomy followed by penetrating keratoplasty. | Chowers I; Ticho U | Excluded | No IOP lowering therapy |
| | Improvement in Vision Parameters for Participants Treated With Alternative Therapies in a 3-day Program. | Kondrot EC | Excluded | No IOP lowering therapy |
| | Ocular side effects associated with peribulbar injections of triamcinolone acetonide for diabetic macular edema. | Chew EY; Glassman AR; Beck RW; Bressler NM; Fish GE; Ferris FL; Kinyoun JL | Excluded | Irrelevant study population |
| | Ibopamine challenge testing becomes negative following successful trabeculectomy surgery. | Landers J; Ullrich K; Craig JE | Excluded | No IOP lowering therapy |
| | Deep sclerectomy with collagen implant: medium term results. | Karlen ME; Sanchez E; Schnyder CC; Sickenberg M; Mermoud A | Excluded | No IOP lowering therapy |
| | Estimating Optical Coherence Tomography Structural Measurement Floors to Improve Detection of Progression in Advanced Glaucoma. | Bowd C; Zangwill LM; Weinreb RN; Medeiros FA; Belghith A | Excluded | No intervention |
| | Latanoprost 0.005% in POAG: effects on IOP and ocular blood flow. | Vetrugno M; Cantatore F; Gigante G; Cardia L | Excluded | Irrelevant outcomes |
| | A 5-year, multicenter, open-label, safety study of adjunctive latanoprost therapy for glaucoma. | Alm A; Schoenfelder J; McDermott J | Excluded | Irrelevant outcomes |
| | Long-term drift and continued efficacy after multiyear timolol therapy. | Steinert RF; Thomas JV; Boger WP 3rd | Excluded | Irrelevant outcomes |
| | Clinical evaluation of the neuroprotective effect of alpha-tocopherol against glaucomatous damage. | Engin KN; Engin G; Kucuksahin H; Oncu M; Guvener B | Excluded | No IOP lowering therapy |
| | [New aspects of corneal pachymetry in congenital glaucoma and pediatric aphakic glaucoma]. | Jordan JF; Dietlein TS; Dinslage S; Krieglstein GK | Excluded | No intervention |
| | Leksell gamma knife treatment of uveal melanoma. | Simonová G; Novotný J Jr; Liscák R; Pilbauer J | Excluded | Irrelevant study population |
| | Phacoemulsification-deep sclerectomy modified by trabeculum microperforations and implantation of lens anterior capsule as autologous scleral implant. | Rekas M; Rudowicz J; Lewczuk K; Kluś A; Pawlik B; Stankiewicz A | Excluded | Irrelevant study population |
| | Prospective evaluation of penetrating deep sclerectomy in advanced open-angle glaucoma: Filtration surgery adapted to resource scarcity in developing countries. | Kalala A; Gillmann K; Mermoud A | Excluded | No intervention |
| | [The effects of corneal cross-linking on intraocular pressure measurement in keratoconus]. | Badri S; Mchachi A; Alnadhari M; Benhmidoune L; Chakib A; Rachid R; Elbelhadji M; Amraoui A | Excluded | No intervention |
| | Long-term results of deep sclerectomy with collagen implant. | Shaarawy T; Mansouri K; Schnyder C; Ravinet E; Achache F; Mermoud A | Excluded | Irrelevant study population |
| | Five-year results of deep sclerectomy with collagen implant. | Shaarawy T; Karlen M; Schnyder C; Achache F; Sanchez E; Mermoud A | Excluded | Irrelevant study population |
| | [Treatment of diabetic macular edema with intravitreal triamcinolone acetonide injection: functional and anatomical outcomes]. | Selim Kocabora M; Kucuksahin H; Gulkilik G; Taskapili M; Yilmazli C; Engin G | Excluded | Irrelevant study population |
| | Mini-trabeculectomy as initial surgery for medically uncontrolled glaucoma. | Ophir A | Excluded | Irrelevant outcomes |
| | [Efficacy of selective laser trabeculoplasty in the treatment of primary open-angle glaucoma]. | Gracner T; Pahor D; Gracner B | Excluded | Irrelevant outcomes |
| | [Long-term follow-up of selective laser trabeculoplasty in primary open-angle glaucoma]. | Gracner T; Falez M; Gracner B; Pahor D | Excluded | Irrelevant outcomes |
| | [Modern assessment of perimetric progression in glaucoma]. | Feraru CI; Pantalon A | Excluded | No intervention |
| | [Digital planimetry for long-term follow-up of glaucomatous optic disk injuries in patients with normal pressure glaucoma]. | Nguyen NX; Meindl C; Horn FK; Dzialach M; Langenbucher A; Jünemann A; Mardin CY | Excluded | No intervention |
| | [Laser trabeculoplasty in glaucoma chronicum simplex. Results of a prospective, clinical study]. | Vogel M; Ecker S; Cieplik G; Bernhardt G | Excluded | Irrelevant study population |
| | [Cataract surgery in patients with end-stage glaucoma]. | Altmeyer M; Wirbelauer C; Häberle H; Pham DT | Excluded | No IOP lowering therapy |
| | Sympathetic use of latanoprost 0.005% in 30 eyes. | Montrone F; Macinagrossa G; Tenerelli F | Excluded | No intervention |
| | [Radial optic neurotomy for severe central retinal vein occlusion: preliminary results]. | Le Rouic JF; Becquet F; Zanlonghi X; Péronnet P; Pousset-Decré C; Hermouet-Leclair E; Ducournau D | Excluded | Irrelevant study population |
| | [Primary trabeculectomy for open-angle glaucoma with mitomycin C]. | Hara T; Shirato S; Miyata N; Eguchi K; Takada M | Excluded | Irrelevant outcomes |
| | Glaucoma Screening With Artificial Intelligence | NCT06012058, | Excluded | No intervention |
| | The Secondary Beneficial Effects of Prostaglandin Analog Treatment in Thyroid Eye Disease Patients | NCT01927406, | Excluded | Irrelevant study population |
| | Prophylaxis for Anti-VEGF-induced IOP Elevation | NCT02623023, | Excluded | No intervention |
| | Canadian Glaucoma Study: 3. Impact of risk factors and intraocular pressure reduction on the rates of visual field change. | Chauhan BC; Mikelberg FS; Artes PH; Balazsi AG; LeBlanc RP; Lesk MR; Nicolela MT; Trope GE | Excluded | Insufficient progression velocity data |
| | Comparison of the effects of dorzolamide/timolol and latanoprost/timolol fixed combinations upon intraocular pressure and progression of visual field damage in primary open-angle glaucoma. | Pajic B; Pajic-Eggspuehler B; Häfliger IO | Excluded | Insufficient progression velocity data |
| | Effect of 0.005% latanoprost once daily on intraocular pressure in glaucomatous patients not adequately controlled by beta-blockers twice daily: a 3-year follow-up. Experience and incidence of side effects in a prospective study on 76 patients. | Costagliola C; Del Prete A; Verolino M; Antinozzi P; Fusco R; Parmeggiani F; Mastropasqua L | Excluded | Irrelevant outcomes |
| | Effect of treatment on the rate of visual field change in the ocular hypertension treatment study observation group | De Moraes, CG; Demirel, S; Gardiner, SK; Liebmann, JM; Cioffi, GA; Ritch, R; Gordon, MO; Kass, MA | Included | |
| | Effects of Brimonidine and Timolol on the Progression of Visual Field Defects in Open-angle Glaucoma: a Single-center Randomized Trial | Yokoyama, Y; Kawasaki, R; Takahashi, H; Maekawa, S; Tsuda, S; Omodaka, K; Nakazawa, T | Excluded | Insufficient progression velocity data |
| | Effect of timolol versus pilocarpine on visual field progression in patients with primary open-angle glaucoma | Vogel, R; Crick, RP; Mills, KB; Reynolds, PM; Sass, W; Clineschmidt, CM; Tipping, R | Excluded | Insufficient progression velocity data |
| | The Effect of Body Posture on Intraocular Pressure in Progressive Glaucoma | NCT01351779 | Excluded | No IOP lowering therapy |
